# Assessing the Accuracy of Artificial Intelligence Chatbots in the Diagnosis and Management of Meniscal Tears

**DOI:** 10.7759/cureus.84124

**Published:** 2025-05-14

**Authors:** Jason S DeFrancisis, Peter Richa, Daniel Oar, Luke Henwood, Zachary J Buchman, Gagan Grewal

**Affiliations:** 1 Orthopedic Surgery, Lake Erie College of Osteopathic Medicine, Bradenton, USA; 2 Physical Medicine and Rehabilitation, Lake Erie College of Osteopathic Medicine, Bradenton, USA; 3 Orthopedic Surgery, Larkin Community Hospital, Miami, USA

**Keywords:** ai chatbot, artificial intelligence (ai), chat gpt-4o, google gemini, meniscal tear, meniscus, meniscus tear, orthopaedic surgery, orthopedic surgery

## Abstract

Introduction: Artificial intelligence (AI) chatbots have emerged as readily accessible tools for providing medical information to the public. However, the accuracy of AI chatbot responses, particularly in specialized medical fields such as orthopaedic surgery, remains largely understudied.

Objective: This study aims to evaluate the accuracy of responses from two prominent free AI chatbots when posed with frequent questions about meniscus tears, a common orthopaedic injury.

Methods: The two AI chatbots assessed in this study were ChatGPT-4o and Gemini 2.0 Flash. The analysis focused on the number of statements provided by each chatbot and the percentage of verifiable statements based on UpToDate alone, as well as UpToDate combined with peer-reviewed articles as of March 2025.

Results: The results showed no statistically significant difference in the average number of statements generated per question between the two AI chatbots. ChatGPT-4o provided an average of 18.25 statements per question, while Gemini 2.0 Flash generated 19.50 statements per question (p>0.05). Similarly, there was no significant difference in the percentage of verifiable statements provided by each AI chatbot. ChatGPT-4o achieved 58.22% verifiable statements compared to Gemini 2.0 Flash’s 58.97% when using UpToDate as the sole verification source, and 83.56% versus 84.62%, respectively, when incorporating both UpToDate and peer-reviewed articles as verification sources (p>0.05). However, a statistically significant difference in the percentage of verifiable statements was observed based on the verification source used. UpToDate alone resulted in 58.61% of verifiable statements, while combining UpToDate and peer-reviewed articles increased this percentage to 84.11% (p<0.0001).

Conclusion: Overall, the results of this study suggest that there are minimal differences between free AI chatbots in providing orthopaedic medical information. The results also emphasize the importance of utilizing broader verification sources to enhance the accuracy of AI-generated statements. The study indicates that AI chatbots have clinical limitations in their accuracy and understanding of specific orthopaedic conditions. The authors suggest that although AI chatbots can contribute to orthopaedic care and patient education, they are not capable of replacing the clinical judgment or expertise of orthopaedic surgeons.

## Introduction

Artificial intelligence (AI) represents the simulation of human intelligence by a system or machine [1]. AI is designed to emulate human thought by integrating perception, reasoning, learning, planning, and prediction to facilitate optimal decision-making [1]. AI possesses the ability to provide an extensive amount of information at a user's fingertips within seconds of a search [2]. A commonly used variation of AI, chatbots, may provide information by answering questions, providing explanations, and offering additional resources [3]. The accessibility and utilization of AI chatbots are increasing in various fields, including health care [3,4]. AI chatbots can be used to facilitate conversations and address the different healthcare needs of potential patients [5]. One way AI chatbots have the potential to provide medical information is through their integration with basic Internet search engines, which is becoming increasingly common [6]. The Internet has long served as a primary source of medical information among adults. A study conducted by Wang and Cohen discovered that over a six-month period, 58.5% of all adults used the Internet to search for health or medical information [7]. The Internet's accuracy in medical advice has always been called into question; however, with the integration of AI chatbots, speculation about improved accuracy is a conversation of clinical importance [6,7].

Among the various AI chatbots, ChatGPT-4o, designed by OpenAI, and Gemini 2.0 Flash, created by Google, are commonly utilized and have become key sources for medical information inquiries [8-10]. Despite the ongoing developments in AI chatbot technology, skepticism remains regarding the accuracy and reliability of the information they provide [8,11]. It has been documented that AI chatbots frequently make factual mistakes and provide imprecise medical information [11]. The growing usage and accessibility of AI chatbots, along with the potential for errors, prompt discussions regarding their accuracy and reliability in providing medical advice to prospective patients [11]. 

In particular, in the field of orthopaedic surgery, AI holds significant potential to transform patient care, offering advancements in diagnosis, treatment planning, and surgical precision [12]. AI chatbots can provide patients with information regarding orthopaedic care, which can improve patient education and offer preliminary guidance [13]. However, it is important to note that while AI chatbots show promise in providing orthopaedic information, their accuracy and consistency can vary [12,13]. The increased usage and potential for inaccurate information regarding orthopaedics have prompted the authors to investigate the accuracy of AI chatbots regarding information on a common orthopaedic pathology, meniscus tears, compared to reputable existing literature [14]. 

Menisci play a crucial role in enhancing the stability of the femorotibial joint [15]. They help distribute axial load, absorb shock, and supply lubrication and nutrition to the knee joint [15]. Meniscus tears have an estimated incidence rate of 60 per 100,000, which is thought to be underestimated [16]. They most commonly affect males [16]. Meniscus tears are most common in young active populations due to acute trauma, but they are also common in the elderly due to degenerative tears [14]. Meniscus tears typically present with joint effusion, localized tenderness along the joint line, and symptoms such as pain, increased instability, and mechanical issues, including sensations of locking, catching, clicking, and popping [17]. Meniscus tears are diagnosed through clinical exams utilizing tests such as McMurray’s, Apley’s, and Thessaly’s in conjunction with imaging such as magnetic resonance imaging (MRI) [18]. MRI is the imaging modality of choice in diagnosing meniscus tears [18]. Meniscus treatment options range from conservative measures to various surgical interventions, including partial meniscectomy, total meniscectomy, meniscus repair, and meniscal replacement [19]. Due to the high prevalence of meniscus tears, meniscus surgery is one of the most common orthopaedic procedures [14]. Given this and the rising use of AI chatbots, the authors believe that investigating the use of AI chatbots for gathering medical information on meniscus tears is a critical area of research.

While current research has investigated the accuracy of AI chatbots in analysing orthopaedic pathology, to the best of the author's knowledge, no comprehensive study has specifically explored the medical information provided by AI chatbots regarding meniscus tears and assessed its accuracy. Existing studies in other medical fields with similar guidelines have inspired the authors to investigate two AI chatbots with questions regarding meniscus tears to look at the validity of responses [20]. This study aims to evaluate the accuracy of statements provided by AI chatbots, ChatGPT-4o and Gemini 2.0 Flash, regarding medical information related to meniscus tears by comparing the statements made by the AI chatbots with UpToDate and published peer-reviewed articles. UpToDate is a widely recognized standard reference that offers evidence-based consensus knowledge derived from the highest quality and most current clinical evidence [21]. It is important to acknowledge the inherent subjectivity involved in the semantic nature of data acquisition, as well as the potential for minor inferences drawn from UpToDate and peer-reviewed articles that may not explicitly support certain statements. Nevertheless, efforts were made to increase the objectivity of this study by utilizing a considerable number of statements recorded across two AI chatbots and incorporating direct quotes from UpToDate and peer-reviewed articles to ensure a solid, reliable foundation for validating the findings. The authors aim to contribute to the growing body of literature on AI in orthopaedic surgery and gain a deeper insight into the accuracy of medical information provided by AI chatbots in response to queries from prospective patients. Given the increasing use and developments of AI, we believe this is an essential area for further investigation.

## Materials and methods

The current research project began on March 1, 2025. The research team presented eight questions, categorized into four domains: general information, symptoms and diagnosis, treatment options, and recovery and prognosis of meniscus tears, to two free AI chatbots: Gemini 2.0 Flash and ChatGPT-4o. The specific questions posed to both AI chatbots are listed in Table 1. To maintain consistency in the responses provided by the AI chatbots, all questions were introduced with the opening statement “In paragraph format,” followed by the respective questions as listed in Table 1. This standardized format was used consistently across both AI chatbots to ensure uniformity in the prompting process.

**Table 1:** Questions presented to chatbots by domain. Questions presented to each chatbot categorized by domain. All questions were prefaced with “In paragraph format” followed by the specific question.

Standardization technique	Domain of prompt	Question
“In paragraph format”	General Information	What is a meniscus tear?
“In paragraph format”	General Information	What causes a meniscus tear?
“In paragraph format”	Symptoms and Diagnosis	What are the symptoms of a torn meniscus?
“In paragraph format”	Symptoms and Diagnosis	How is a meniscus tear diagnosed?
“In paragraph format”	Treatment Options	How is a meniscus tear treated?
“In paragraph format”	Treatment Options	Will I need surgery for my meniscus tear?
“In paragraph format”	Recovery and Prognosis	What is the recovery time for a meniscus tear?
“In paragraph format”	Recovery and Prognosis	What are the long-term complications of an untreated torn meniscus?

As the questions outlined in Table 1 were prompted to the AI chatbots, both AI chatbots provided a complete paragraph response to each question. A total of 16 complete paragraph responses were generated, with ChatGPT-4o and Gemini 2.0 Flash each providing eight responses. Subsequently, the full paragraph responses were compiled into a centralized spreadsheet for comprehensive analysis. The research team systematically identified and extracted “Statements Presented as Factual” (SPAF), defined as components of the AI chatbot answers that can be corroborated as factual statements, as described by Buchman et al. [20]. An example of SPAF extraction can be found in Table 2.

**Table 2:** A demonstration of SPAF extraction. An example of SPAF extraction from a snippet of a provided answer. Individual SPAF are indicated by superscript numbers in the response text, correlated to the numbered list on the right. SPAF: statements presented as factual.

Question: Snippet from “What is a meniscus Tear?”		Extracted SPAF
Symptoms of a meniscus tear include pain^1^, swelling^2^, stiffness^3^, and difficulty moving the knee^4^, sometimes accompanied by a locking or catching sensation^5^.		1 - Symptoms of a meniscus tear include pain.
		2 - Symptoms of a meniscus tear include swelling.
		3 - Symptoms of a meniscus tear include stiffness.
		4 - Symptoms of a meniscus tear include difficulty moving the knee.
		5 - Symptoms of a meniscus tear are sometimes accompanied by a locking or catching sensation

To minimize bias and standardize the extraction process, a predefined set of rules and criteria was established, as shown in Table 3. To ensure consistency in data extraction and minimize variability, one team member was assigned to handle all data extraction and isolation of individual factual statements. An intra-observer and inter-observer comparison was made to validate the collected data. This approach was employed to mitigate potential discrepancies that could arise if multiple researchers independently interpreted and extracted the data.

**Table 3:** Predefined criteria utilized for SPAF extraction. The standard rules were followed by the research team during the process to facilitate more refined SPAF extractions. The standard rules are accompanied by corresponding examples and explanations. SPAF: statements presented as factual.

Standard rule	Example response	Explanation
For responses containing “and,” each component of the statement is treated as a distinct SPAF and is analysed independently during the verification process.	“Physical therapy is often recommended to strengthen the muscles around the knee and improve stability.”	In this response, there are two SPAF: 1-Physical therapy is often recommended to strengthen the muscles around the knee 2-Physical therapy is often recommended to improve stability
For responses containing “or,” the entire statement is considered as a single factual statement. During the verification process, confirmation of either component was deemed sufficient to classify the entire statement as supported.	“The torn meniscus can cause recurrent episodes of knee locking or catching.”	In this response, there is one SPAF, and if either “meniscus can cause recurrent episodes of knee locking” or “meniscus can cause recurrent episodes of knee catching” can be verified, the SPAF is considered supported.

Following the extraction of all SPAF, each assertion underwent manual accuracy verification. The primary medical reference selected was UpToDate (Wolters Kluwer) as of March 2025 [21]. UpToDate was selected as the primary reference, as it is a well-established standard reference that offers evidence-based information and guidelines. To avoid publication bias that may be present in UpToDate, a secondary medical reference was used, which was any peer-reviewed article related to the specific SPAF topic in the Google Scholar database. 

Given the use of two medical references, it was essential to standardize the verification process. Initially, members of the research team cross-checked each SPAF against UpToDate. If the individual SPAF was not supported by UpToDate, a broader search of peer-reviewed articles was conducted. When a SPAF was designated as “Supported” by UpToDate, the specific section containing the supporting evidence was recorded, along with the corresponding direct quotation for reference. If the SPAF was not designated as “Supported” by UpToDate but was designated as “Supported” by peer-reviewed articles, the specific article title was documented, and the corresponding direct quotation was noted for reference. If a SPAF was designated as “Not Supported” by neither UpToDate nor peer-reviewed articles due to contradicting information, the refuting section or article, along with the direct quotation, was documented. If a SPAF was neither supported nor had a direct contraindication in either UpToDate or by peer-reviewed articles, no quotation was documented, and the SPAF was labelled “Not Supported.” Appropriate author commentary was provided as necessary to add further clarifications. To minimize error and maintain the highest level of standardization, at least one additional member of the research team independently reviewed each entry. Any discrepancies identified between the initial reviewer and the second reviewer were subsequently adjudicated through unanimous consensus among all members of the research team. Each SPAF that underwent verification was compiled into an in-depth document containing the complete paragraph response to each question, the extracted SPAF, its designation as either "Supported" or "Not Supported," along with the corresponding source of evidence, direct quotations, and pertinent author commentary.

After establishing the support status of each SPAF, data were generated and categorized. Two separate sets of data were created, displaying the total amount of SPAF from each AI chatbot and domain, along with the quantity of “Supported” versus “Not Supported” SPAF. A two-tailed, two-sample t-test was conducted to compare the average number of SPAF generated per prompt between the two AI chatbots to assess if there was a statistically significant difference.

To examine the differences in verifiable accuracy between the two AI chatbots, a binary code system was utilized. Each SPAF deemed as “Supported” was labelled with “1,” and those identified as “Not Supported” received a “0.” Two-tailed, two-sample t-tests were created to analyse the differences in the percentage of verifiable SPAF across all prompts for both AI chatbots, first using UpToDate as the sole reference and then using a combined reference set of both UpToDate and peer-reviewed articles. Similarly, two-tailed, two-sample t-tests were performed to analyse the differences between the AI chatbots for each specific domain, using UpToDate as the sole reference, followed by utilizing a combination of both UpToDate and peer-reviewed articles. Finally, a two-tailed, two-sample t-test was conducted to investigate the total number of verifiable SPAF using solely UpToDate versus a combination of both UpToDate and peer-reviewed articles.

These analyses provided valuable comparative assessments of the AI chatbots, examining (1) the average amount of information provided per prompt; (2) the proportion of information supported solely by UpToDate versus UpToDate in addition to peer-reviewed articles for each chatbot; (3) potential variations in chatbot performance overall and specific to the domain of the prompt; (4) potential differences in chatbot performance when using different verification methods (UpToDate only vs UpToDate + peer-reviewed articles).

## Results

**Figure 1:**
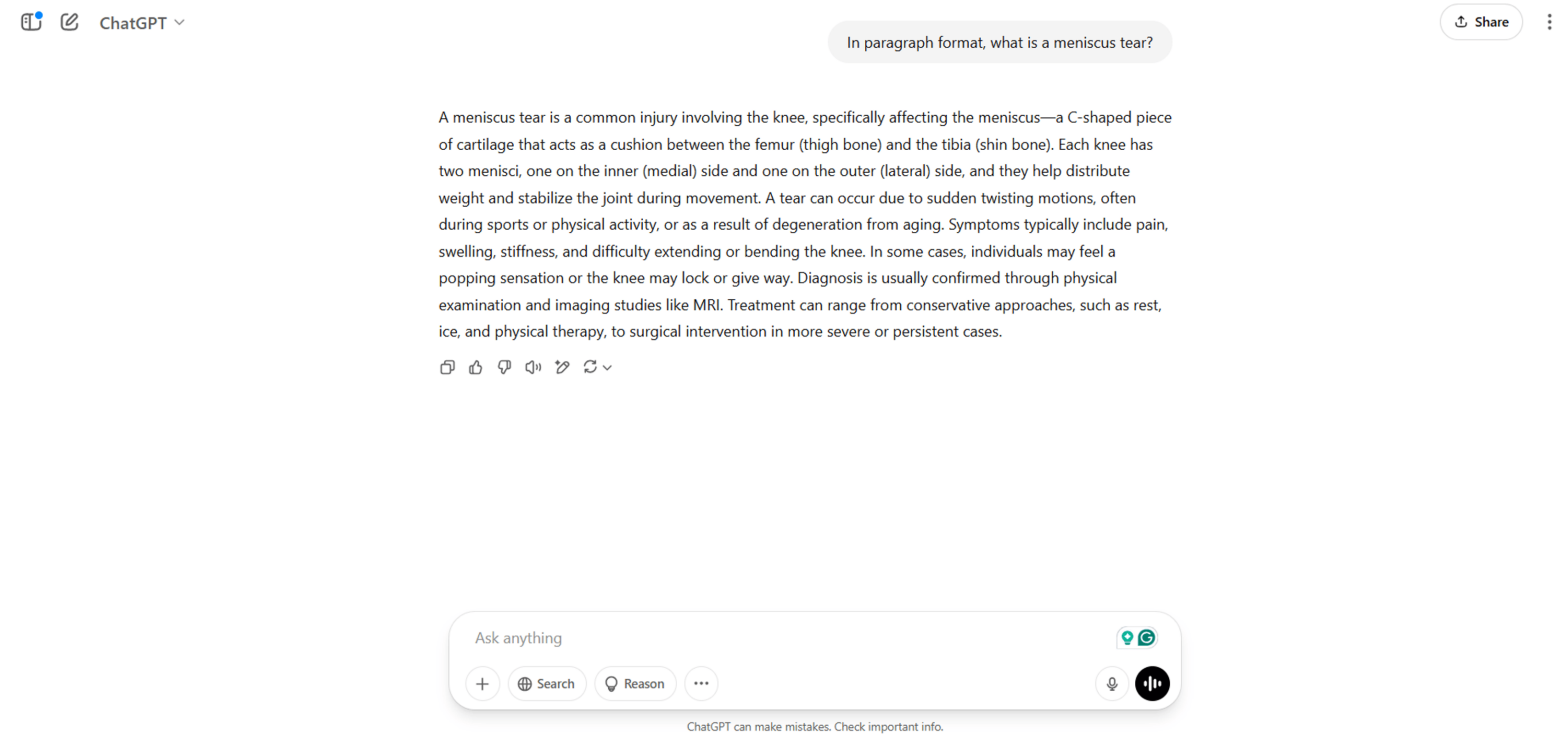
Display of the ChatGPT-4o interface with an example question.

**Figure 2:**
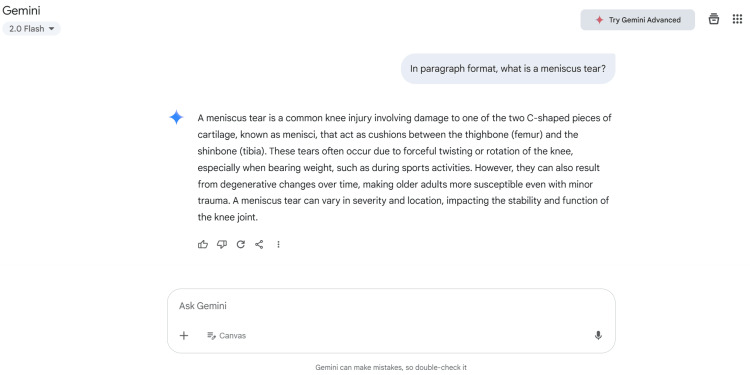
Display of the Gemini 2.0 Flash interface with an example question.

To demonstrate how the questions posed to the different AI chatbots were displayed, we have provided examples. Figure 1 shows the user interface of ChatGPT-4o, and Figure 2 presents the user interface of Gemini 2.0 Flash.

Table 4 displays a summary of data obtained using UpToDate as the only reference. In total, 302 SPAF were extracted from responses of the two AI chatbots to all 16 questions. A total of 177 SPAF were able to be verified with supporting evidence using UpToDate as the only reference (58.97%). A detailed breakdown of the quantity and verifiability of SPAF across different question domains is also displayed in Table 4.

**Table 4:** Summary of data using UpToDate as the only reference. A table presenting the data collected following the SPAF extraction and verification process using UpToDate as the only reference. SPAF: statements presented as factual.

Group	No. of SPAF	No. of SPAF able to be verified	No. of SPAF unable to be verified
ChatGPT-4o (All Domains of Prompts)	146	85	61
ChatGPT-4o (Domain: General Questions)	35	19	16
ChatGPT-4o (Domain: Symptoms & Diagnosis)	34	24	10
ChatGPT-4o (Domain: Treatment Options)	38	32	6
ChatGPT-4o (Domain: Recovery & Prognosis)	39	10	29
Gemini 2.0 Flash (All Domains of Prompts)	156	92	64
Gemini 2.0 Flash (Domain: General Questions)	34	23	11
Gemini 2.0 Flash (Domain: Symptoms & Diagnosis)	37	20	17
Gemini 2.0 Flash (Domain: Treatment Options)	44	34	10
Gemini 2.0 Flash (Domain: Recovery & Prognosis)	41	15	26
Total	302	177	125

Table 5 demonstrates a summary of data obtained using UpToDate and peer-reviewed articles as the reference. In total, 302 SPAF were extracted from responses of the two AI chatbots to all 16 questions. A total of 254 SPAF were able to be verified with supporting evidence using UpToDate and peer-reviewed articles as the reference (84.11%). A detailed breakdown of the quantity and verifiability of SPAF across different question domains is also displayed in Table 5.

**Table 5:** Summary of data using UpToDate and peer-reviewed articles as the reference. A table presenting the data collected following the SPAF extraction and verification process using UpToDate and peer-reviewed articles as the reference. SPAF: statements presented as factual.

Group	No. of SPAF	No. of SPAF able to be verified	No. of SPAF unable to be verified
ChatGPT-4o (All Domains of Prompts)	146	122	24
ChatGPT-4o (Domain: General Questions)	35	30	5
ChatGPT-4o (Domain: Symptoms & Diagnosis)	34	29	5
ChatGPT-4o (Domain: Treatment Options)	38	36	2
ChatGPT-4o (Domain: Recovery & Prognosis)	39	27	12
Gemini 2.0 Flash (All Domains of Prompts)	156	132	24
Gemini 2.0 Flash (Domain: General Questions)	34	30	4
Gemini 2.0 Flash (Domains: Symptoms & Diagnosis)	37	29	8
Gemini 2.0 Flash (Domains: Treatment Options)	44	40	4
Gemini 2.0 Flash (Domains: Recovery & Prognosis)	41	33	8
Total	302	254	48

Table 6 displays the results of a two-tailed, two-sample t-test comparing the mean number of SPAF generated by two AI chatbots across 16 total questions. The results of the t-test revealed no statistically significant difference in terms of the average number of SPAF produced between the two AI chatbots, with a p-value of 0.27. ChatGPT-4o produced an average of 18.25 SPAF per question, while Gemini 2.0 Flash produced an average of 19.50 SPAF per prompt. Although a minor numerical difference of 1.25 was observed, it is not statistically significant, suggesting comparable SPAF generation between ChatGPT-4o and Gemini 2.0 Flash for the 16 total questions posed. 

**Table 6:** Results of a two-tailed, two-sample t-test comparing ChatGPT-4o vs. Gemini 2.0 Flash for average number of SPAF generated per question. SPAF: statements presented as factual.

Chatbot	Average number of SPAF per prompt	Standard deviation	Difference	T-statistic	p-value
GPT-4o	18.25	1.49	1.25	1.51	0.27
Gemini	19.50	2.67			

Tables 7, 8 display the results of two-tailed, two-sample t-tests comparing the percentages of verifiable SPAF across all question domains using UpToDate alone and UpToDate in addition to peer-reviewed articles as reference sources, respectively. Table 7 presents results when using only UpToDate for verification. ChatGPT-4o attained 58.22% SPAF verification, while Gemini 2.0 Flash achieved 58.97% SPAF verification. The minor difference of 0.75% was not statistically significant with a p-value of 0.89, suggesting comparable accuracy between the two AI chatbots when using UpToDate alone for verification. Table 8 presents the results of expanding the verification source to include both UpToDate and peer-reviewed articles. This expansion resulted in higher verification rates for both AI chatbots. ChatGPT-4o achieved 83.56% SPAF verification, while Gemini 2.0 Flash achieved 84.62% SPAF verification. Despite a slight difference of 1.06%, this difference also did not reach statistical significance with a p-value of 0.80, indicating similar SPAF accuracy between ChatGPT-4o and Gemini 2.0 Flash when using UpToDate in conjunction with peer-reviewed articles for SPAF verification. 

**Table 7:** Results of a two-tailed, two-sample t-test comparing ChatGPT-4o vs. Gemini 2.0 Flash for total percent of SPAF able to be verified by only UpToDate (all domains combined). SPAF: statements presented as factual.

Chatbot	Percent of SPAF able to be verified	Variance	Difference	T-statistic	p-value
GPT-4o	58.22	24.49%	0.75%	0.15	0.89
Gemini	58.97	24.35%			

**Table 8:** Results of a two-tailed, two-sample t-test comparing ChatGPT-4o vs. Gemini 2.0 Flash for total percent of SPAF able to be verified by UpToDate + peer-reviewed articles (all domains combined). SPAF: statements presented as factual.

Chatbot	Percent of SPAF able to be verified	Variance	Difference	T-statistic	p-value
GPT-4o	83.56	13.83%	1.06%	0.28	0.80
Gemini	84.62	13.10%			

Tables 9, 10 display the results of two-tailed two-sample t-tests comparing the percentages of verifiable SPAF across the general information domain using UpToDate alone and UpToDate in conjunction with peer-reviewed articles as reference sources, respectively. Table 9 presents the results when using only UpToDate for verification. ChatGPT-4o only achieved 54.29% SPAF verification, while Gemini 2.0 Flash achieved 67.65%. The difference of 13.36% was not statistically significant, with a p-value of 0.26, suggesting comparable accuracy between the two AI chatbots when using UpToDate alone for verification. Table 10 presents the results of expanding the verification source to include both UpToDate and peer-reviewed articles. This expansion resulted in higher verification rates for both AI chatbots. ChatGPT-4o achieved 85.71% SPAF verification, while Gemini 2.0 Flash reached 88.24% SPAF verification. Despite a slight difference of 2.53%, this difference also did not reach statistical significance with a p-value of 0.76, indicating similar SPAF accuracy between ChatGPT-4o and Gemini 2.0 Flash when using UpToDate in addition to peer-reviewed articles for SPAF verification.

**Table 9:** Results of a two-tailed, two-sample t-test comparing ChatGPT-4o vs. Gemini 2.0 Flash for total percent of SPAF able to be verified by only UpToDate (general information domain only). SPAF: statements presented as factual.

Chatbot	Percent of SPAF able to be verified	Variance	Difference	T-statistic	p-value
GPT-4o	54.29	25.55%	13.36%	1.55	0.26
Gemini	67.65	22.55%			

**Table 10:** Results of a two-tailed, two-sample t-test comparing ChatGPT-4o vs. Gemini 2.0 Flash for total percent of SPAF able to be verified by UpToDate + peer-reviewed articles (general information domain only). SPAF: statements presented as factual.

Chatbot	Percent of SPAF able to be verified	Variance	Difference	T-statistic	p-value
GPT-4o	85.71	12.61%	2.53%	0.35	0.76
Gemini	88.24	10.70%			

Tables 11, 12 display the results of two-tailed, two-sample t-tests comparing the percentages of verifiable SPAF across the symptoms and diagnosis domain using UpToDate alone and UpToDate in addition to peer-reviewed articles as reference sources, respectively. Table 11 presents the results when using UpToDate by itself for verification. ChatGPT-4o attained 70.59% SPAF verification, while Gemini 2.0 Flash only achieved a 54.05% verification rate. The difference of 16.53% was not statistically significant with a p-value of 0.16, suggesting comparable accuracy between the two AI chatbots when only using UpToDate for verification. Table 12 displays the results of expanding the verification source to include both UpToDate and peer-reviewed articles. This expansion resulted in higher verification rates for both AI chatbots. ChatGPT-4o achieved 85.29% SPAF verification, with Gemini 2.0 Flash reaching only 78.38% SPAF verification. Despite a difference of 6.92%, this difference again did not reach statistical significance with a p-value of 0.46, indicating similar SPAF accuracy between ChatGPT-4o and Gemini 2.0 Flash when using both UpToDate and peer-reviewed articles.

**Table 11:** Results of a two-tailed, two-sample t-test comparing ChatGPT-4o vs Gemini 2.0 Flash for total percent of SPAF able to be verified by only UpToDate (symptoms & diagnosis domain only). SPAF: statements presented as factual.

Chatbot	Percent of SPAF able to be verified	Variance	Difference	T-statistic	p-value
GPT-4o	70.59	21.39%	16.53%	2.23	0.16
Gemini	54.05	25.53%			

**Table 12:** Results of a two-tailed, two-sample t-test comparing ChatGPT-4o vs Gemini 2.0 Flash for total percent of SPAF able to be verified by UpToDate + peer-reviewed articles (symptoms & diagnosis domain only). SPAF: statements presented as factual.

Chatbot	Percent of SPAF able to be verified	Variance	Difference	T-statistic	p-value
GPT-4o	85.29	12.92%	6.92%	0.91	0.46
Gemini	78.38	17.42%			

Tables 13, 14 present the results of two-tailed, two-sample t-tests comparing the percentages of verifiable SPAF across the treatment domain, using UpToDate alone and UpToDate with peer-reviewed articles as reference sources, respectively. Table 13 presents the results when using only UpToDate for verification. ChatGPT-4o attained 84.21% SPAF verification, while Gemini 2.0 Flash achieved only 77.27% SPAF verification. The difference of 6.94% was again not statistically significant, with a p-value of 0.44, suggesting comparable accuracy between the two AI chatbots when using UpToDate alone. Table 14 presents the results of expanding the verification source to include both UpToDate and peer-reviewed articles. This expansion resulted in higher verification rates for both AI chatbots. ChatGPT-4o achieved an impressive 94.74% SPAF verification rate, while Gemini 2.0 Flash reached a 90.91% SPAF verification rate. Despite a small difference of 3.83%, this difference also did not reach statistical significance with a p-value of 0.51, indicating similar SPAF accuracy between ChatGPT-4o and Gemini 2.0 Flash when using UpToDate and peer-reviewed articles together.

**Table 13:** Results of a two-tailed, two-sample t-test comparing ChatGPT-4o vs. Gemini 2.0 Flash for total percent of SPAF able to be verified by only UpToDate (treatment options domain only). SPAF: statements presented as factual.

Chatbot	Percent of SPAF able to be verified	Variance	Difference	T-statistic	p-value
GPT-4o	84.21	13.66%	6.94%	0.97	0.44
Gemini	77.27	17.97%			

**Table 14:** Results of a two-tailed, two-sample t-test comparing ChatGPT-4o vs. Gemini 2.0 Flash for total percent of SPAF able to be verified by UpToDate + peer-reviewed articles (treatment domain only). SPAF: statements presented as factual.

Chatbot	Percent of SPAF able to be verified	Variance	Difference	T-statistic	p-value
GPT-4o	94.74	5.12%	3.83%	0.79	0.51
Gemini	90.91	8.46%			

Tables 15, 16 display the results of two-tailed two-sample t-tests comparing the percentages of verifiable SPAF across the recovery and prognosis domain using UpToDate alone and UpToDate along with peer-reviewed articles as reference sources, respectively. Table 15 presents the results when using UpToDate as a standalone source for verification. ChatGPT-4o attained only a mere 25.64% SPAF verification, while Gemini 2.0 Flash achieved only 36.59%. The difference of 10.94% was not statistically significant with a p-value of 0.30, suggesting comparable accuracy between the two AI chatbots when using UpToDate as a standalone source. Table 16 presents the results of expanding the verification source to include both UpToDate in addition to peer-reviewed articles. This expansion resulted in higher verification rates for both AI chatbots. ChatGPT-4o achieved 69.23% SPAF verification, while Gemini 2.0 Flash reached 80.49% SPAF verification. Despite a small difference of 11.26%, this difference again did not reach statistical significance with a p-value of 0.25, indicating similar SPAF accuracy between ChatGPT-4o and Gemini 2.0 Flash when using both UpToDate and peer-reviewed articles for SPAF verifications.

**Table 15:** Results of a two-tailed, two-sample t-test comparing ChatGPT-4o vs. Gemini 2.0 Flash for total percent of SPAF able to be verified by only UpToDate (recovery and prognosis domain only). SPAF: statements presented as factual.

Chatbot	Percent of SPAF able to be verified	Variance	Difference	T-statistic	p-value
GPT-4o	25.64	19.57%	10.94%	1.40	0.30
Gemini	36.59	23.78%			

**Table 16:** Results of a two-tailed, two-sample t-test comparing ChatGPT-4o vs. Gemini 2.0 Flash for total percent of SPAF able to be verified by UpToDate + peer-reviewed articles (recovery and prognosis domain only). SPAF: statements presented as factual.

Chatbot	Percent of SPAF able to be verified	Variance	Difference	T-statistic	p-value
GPT-4o	69.23	21.86%	11.26%	1.60	0.25
Gemini	80.49	16.10%			

Table 17 displays a comparative analysis of verification methods for SPAF using a two-tailed, two-sample t-test comparing the total percentage of verifiable SPAF across both AI chatbots combined. The analysis compared verification rates between UpToDate alone and UpToDate with peer-reviewed articles. This analysis considered all domains included in the study across both ChatGPT-4o and Google Gemini 2.0 Flash together. A statistically significant difference was observed between the two verification methods (p<0.0001). Verification using UpToDate alone produced a mean of only 58.61% verifiable SPAF, while the combined use of UpToDate and peer-reviewed articles led to a mean of 84.11% verifiable SPAF, with a difference of 25.20%. The findings suggest that incorporating a wider range of verification sources significantly improves SPAF verification rates, demonstrating the value of comprehensive reference sources for validating AI chatbot-generated content.

**Table 17:** Results of a two-tailed, two-sample t-test comparing total percent of SPAF able to be verified using UpToDate alone vs. UpToDate + peer-reviewed articles. SPAF: statements presented as factual.

Reference method	Percent of SPAF able to be verified	Variance	Difference	T-statistic	p-value
UpToDate alone	58.61	24.34%	25.50%	31622.77	0.000000001
UpToDate + peer reviewed articles	84.11	13.41%			

Summary of significant findings

1. There was no significant difference in the number of verifiable SPAF produced per question between ChatGPT-4o and Gemini 2.0 Flash (p>0.05).

2. There was no significant difference in the percentage of verifiable SPAF using any verification method between ChatGPT-4o and Gemini 2.0 Flash (all p>0.05).

3. There was a significant difference in the percentage of verifiable SPAF using UpToDate alone vs. UpToDate + peer-reviewed articles (p<0.0001*), with significantly more SPAF able to be verified when using UpToDate in addition to peer-reviewed articles for verification.

## Discussion

This study evaluated the accuracy of two AI chatbots, ChatGPT-4o and Gemini 2.0 Flash, regarding medical information on a common orthopaedic pathology: meniscus tears. After prompting each chatbot with eight individual questions spanning four separate domains, we concluded that there was no significant difference between ChatGPT-4o and Gemini 2.0 Flash in terms of the amount of SPAF provided (p>0.05) or the percentage of verifiably accurate SPAF (p>0.05) provided. However, a significant difference was observed in the percentage of verifiable information when comparing the use of UpToDate alone versus UpToDate in conjunction with peer-reviewed articles as the verification source (p<0.0001).

The results of this study suggest no significant difference between ChatGPT-4o and Gemini 2.0 Flash in providing medically accurate information on meniscus tears. This suggests that there is likely a negligible difference when selecting an AI chatbot to receive clinically accurate information regarding general information, symptoms, diagnosis, treatment options, recovery, and prognosis of meniscal tears. The minor variation in information quantity and accuracy may be attributed to both AI chatbots being publicly accessible at no cost [9,10]. Since neither chatbot requires a subscription to access and use unrestricted, they likely have a comparable ability to provide reasonably accurate responses. If a paid, subscription-based AI chatbot had been included in the study, there may have been a significant difference in the quantity and accuracy of generated responses. Additionally, neither AI chatbot utilized in this study is designed specifically for medical use. Both AI chatbots are general-purpose AI tools without a specialized focus on providing medical information [9,10]. An AI chatbot specifically designed to provide high-quality, medically accurate information may provide more comprehensive and precise responses. Overall, the combined accuracy of both AI chatbots, when verified against UpToDate and peer-reviewed articles, was 84.11% (Table 17). This accuracy rate is relatively high; however, it remains insufficient to consistently rely on for clinical decision-making, as it does not account for inter-patient variability. Overall, AI chatbots showed limitations in providing accurate and complete information about meniscus tears. Previous research has similar findings; a study conducted by Au Yeung et al. determined that AI chatbots, despite providing highly relevant information, are not yet suitable for clinical use due to key omissions in diagnostic information [22]. Au Yeung et al. also found that AI chatbots tend to provide superficial rather than detailed disease-specific information needed for optimal clinical judgment [22].

In addition to investigating the overall accuracy of each AI chatbot, and both AI chatbots together, this study analysed the accuracy of the AI chatbots in regard to specific domains, including “General information,” “Symptoms and Diagnosis,” “Treatment Options,” and “Recovery and Prognosis.” It was discovered that the “Treatment Options” domain had the highest accuracy, and the “Recovery and Prognosis” domain had the lowest accuracy. This finding was consistent in both AI chatbots and both methods of statement verification. This discrepancy in accuracy may stem from the differences in complexity and variability of the two topic categories. The treatment of meniscus tears is well-documented and well-established in the literature and generally falls into three broad categories: non-operative management, meniscectomy, and meniscus repair [19,23]. Due to the well-established treatment guidelines, the AI chatbots may have been able to generate more accurate responses [23]. In contrast, the recovery and prognosis of meniscus tears are inherently more variable and subjective in nature [23]. Multiple factors influence postoperative outcomes, including tear characteristics, tear location, vascular supply, and the selected treatment approach [23]. Patient-specific factors such as age, comorbidities, and adherence to postoperative management also contribute to variability in recovery [23]. Additionally, there is no single consensus regarding postoperative rehabilitation strategies for meniscus tears [24]. Rehabilitation protocols vary based on tear type and patient-specific considerations [24]. Given the lack of consensus and high dependence on individualized patient factors, the AI chatbots may have struggled to generate responses with the same level of accuracy as they did for treatment-related questions.

In the field of orthopaedics specifically, previous research has demonstrated that AI chatbots provide inconsistent information on common orthopaedic conditions [25]. A study by Khabaz et al. found that AI chatbots have limitations in factual accuracy and completeness when providing orthopaedic information [26]. Additionally, Khabaz et al. highlighted that AI chatbots may be inaccessible to populations with lower health literacy, which can further restrict their utility as a patient education tool [26]. While AI has the potential to assist in orthopaedic surgeries, improve patient outcomes, and reduce physician workload, clinical applications of AI chatbots remain limited [13,27].

A key finding of this study was that the type of verification method utilized had a significant effect on the rate of verifiable SPAF. In particular, a broader scope of reference that included both UpToDate and peer-reviewed articles was able to successfully verify more SPAF compared to using UpToDate alone. This has important implications for future studies on the usage of AI chatbots in orthopaedic conditions, as it suggests a potential bias in UpToDate regarding orthopaedic information. UpToDate may, in some circumstances, fail to verify a SPAF even when that statement is indeed factually correct. In future studies, to ensure fair verification of AI-generated medical content, the authors recommend using a large reference database with the inclusion of peer-reviewed literature. Given that AI chatbots synthesize information from a wide range of sources, a similarly broad verification approach should be utilized for a fair assessment of their accuracy [6]. 

Although this study provides valuable insights, it has limitations. Limitations include a small sample size of questions and SPAF as well as the subjective nature of SPAF extraction and verification. A small sample size can lead to less reliable conclusions, and thus, the findings of this study should be interpreted with caution [28]. In the future, a greater quantity and wider range of questions should be posed to AI chatbots in an attempt to further assess a greater number of SPAF for accuracy. By doing this, more concrete conclusions can be derived from these larger and broader sample sizes [28]. Another limitation of the study is the inherent subjectivity in SPAF extraction and verification. While a standardized approach was followed, including standardized extraction and verification, strict guidelines, and multiple author verification to minimize bias, future research may benefit from further refining this verification process. One potential improvement could involve assessing AI chatbots’ knowledge of common orthopaedic conditions through clinical vignettes. By assessing the AI chatbots’ orthopaedic knowledge in this manner, we can draw further conclusions on the AI chatbots’ clinical judgment and diagnostic skills by simulating a real patient interaction. Additionally, verifying chatbot responses against a broader standardized reference source may further improve the accuracy of SPAF verification. Although the authors believe the measures taken in this study were sufficient to mitigate bias and subjectivity, research methodologies should continue to evolve. Future studies should explore alternative assessment methods, such as clinical vignette-based evaluation, to gauge the reliability and accuracy of AI chatbots in a more clinical setting. Future research on this topic is essential because AI chatbots are being used more frequently than ever before. Additionally, they are projected to expand in their accuracy and capabilities over time. Thus, it is important to monitor how the rapidly growing field of AI can impact orthopaedics and medicine as a whole in the future [29].

Based on the findings of this study and existing literature, the authors suggest that although AI chatbots may have a role in providing patient education and can help overcome one set of referrals from primary care to orthopaedic surgery, thereby saving time and providing adequate directions to treat the condition. However, AI chatbots are not capable of replacing the clinical judgment or expertise of experienced orthopaedic surgeons. AI chatbots often fail to provide fully accurate, comprehensive information and are currently incapable of making nuanced clinical judgements with specific disease and patient information in mind. AI chatbots can be used as a reasonably reliable source of information in some situations but should not be used as an independent source and should not replace a comprehensive and individualized appointment with an orthopaedic surgeon.

## Conclusions

This study provides valuable information on the performance of AI chatbots in providing medical information on meniscus tears. The findings suggest that ChatGPT-4o and Gemini 2.0 Flash demonstrate similar performance with no significant difference in the quantity of information provided or the percentage of information verified as factual. This study highlights limitations in the accuracy of AI chatbots in providing orthopaedic medical information, with the AI chatbots having a combined accuracy of 84.11% when verified against UpToDate and peer-reviewed articles as a combined reference source. The accuracy level, while relatively high, remains insufficient for reliable medical decision-making and patient education purposes.

Overall, the results of this study indicate that AI chatbots, despite their potential, are not yet suitable for orthopaedic clinical use due to limitations in their accuracy and understanding of specific patient and disease processes. The observed discrepancy in accuracy across different domains of meniscus tear information highlights the challenges AI chatbots face in addressing complex orthopaedic topics. The authors suggest that these findings emphasize the continued importance of professional human expertise in orthopaedic care and that while AI chatbots can serve as initial information sources, they should not replace a formal consultation with an orthopaedic surgeon.
